# Serum lipidome remodeling in viral pneumonia: from pathophysiology to therapeutics

**DOI:** 10.3389/fimmu.2026.1836315

**Published:** 2026-06-22

**Authors:** Yige Wang, Qiang Xiao

**Affiliations:** 1 Second Clinical Medical College, Nanjing Medical University, Nanjing, Jiangsu, China; 2 Department of Pulmonary and Critical Care Medicine, Changde Hospital, Xiangya School of Medicine, Central South University (The First People’s Hospital of Changde City), Hunan, China

**Keywords:** ARDS, ferroptosis, HDL dysfunction, Host-directed therapy, immunometabolism, lipidomics, SREBP pathway, viral pneumonia

## Abstract

Viral pneumonia remains a preeminent global health threat, frequently culminating in acute respiratory distress syndrome (ARDS) and systemic organ failure. While traditional paradigms have centered on protein-based inflammatory cascades, this review synthesizes a vast body of emerging evidence to redefine viral pneumonia as a profound systemic metabolic crisis, specifically characterized by the radical remodeling of the host lipidome. Based on a meta-synthesis of clinical and mechanistic data, we delineate a consistent “metabolic crash” during severe infection, where a precipitous decline in serum low-density lipoprotein (LDL)-cholesterol and the functional conversion of high-density lipoprotein (HDL) from a protective “immunometabolic shield” into a pro-inflammatory vehicle serve as decisive prognostic indicators. We further dissect the molecular mechanisms of this reprogramming, detailing how respiratory viruses hijack the host SREBP-SCAP axis to repurpose lipid droplets for viral assembly, while simultaneously triggering ferroptotic cell death through the exhaustion of the GPX4-lipid-peroxidase defense system. By integrating the “gut-lipid-lung axis” and the role of systemic metainflammation, we illustrate how the host’s baseline metabolic architecture dictates the threshold for lethal alveolar-capillary barrier failure. Finally, we evaluate the therapeutic potential of restoring lipid homeostasis through specialized pro-resolving mediators (SPMs) and metabolic stabilizers. We conclude that transitioning toward a lipid-centric precision medicine model, supported by AI-driven metabolic endotyping, is essential for advancing host-directed therapies in the management of severe respiratory viral infections.

## 1 Introduction

Viral pneumonia is one of the leading global health threats with high incidence and serious complications such as acute respiratory distress syndrome (ARDS) (1, 2). From the long-term challenge posed by influenza A virus (IAV) to the unprecedented global disruption caused by the SARS-CoV-2 pandemic, respiratory viruses keep evolving to escape host defense mechanisms (3, 4). While early studies have long focused on the “cytokine storm” as the main cause of lung lesions, recent progress in immunometabolism has turned attention to the systemic and cellular metabolic profile, especially the extensive remodeling of lipid metabolism (5, 6).

Lipids are no longer seen as inert structural components or simple energy reserves. They are now known to act as important regulators in viral entry, replication and the host inflammatory response (7, 8). In the early stages of infection, viruses rely on lipid rafts (cholesterol-rich membrane microdomains) on the host cell surface to help viral fusion and endocytosis with the host cell (9). As the disease progresses, high-throughput omics technologies have revealed a systemic “lipidomic storm” that drastically changes the serum lipid profile (10, 11). A hallmark of this metabolic disorder is a marked decrease in high-density lipoprotein cholesterol (HDL-C) and apolipoprotein A-1 (ApoA-I), both of which are independent predictors of disease severity and mortality in hospitalized patients (12, 13).

In addition, the lung is a lipid−centric organ where surfactant homeostasis is critical for preserving alveolar integrity. Damage to the alveolar-capillary barrier allows circulating lipids and metabolic products to interact with the pulmonary environment, thus activating lethal pathways such as ferroptosis—a form of regulated cell death driven by excessive lipid peroxidation and iron dependence (14, 15). This systemic–local crosstalk is also regulated by the “gut–lipid–lung axis”, in which microbial metabolites affect the types of circulating lipids and further influence the inflammatory status of the lungs (16, 17). Although a large amount of data has been accumulated, a comprehensive overview linking clinical prognosis and molecular pathogenesis through serum lipid remodeling remains lacking.

This review provides a multidimensional analysis of lipidomic changes in viral pneumonia by integrating recent clinical and mechanistic evidence. We discuss the diagnostic value of serum lipid signatures, dissect the mechanisms of viral-mediated lipid hijacking and ferroptosis, and evaluate the therapeutic potential of targeting lipid pathways, such as through specialized pro-resolving mediators (SPMs) and statins (18, 19).

## 2 Clinical lipidomic signatures of viral pneumonia: a multi-omics landscape

### 2.1 The systemic “metabolic crash”: quantitative trajectories and stratification of severity

Severe viral pneumonia—especially when caused by highly pathogenic influenza or coronaviruses—follows a clinical course marked by rapid, dramatic shifts in systemic metabolism. Recent high-impact clinical metabolomics research has coined this phenomenon the “lipid crash,” and it has been well-documented in numerous studies (20, 21). Circulating lipid concentrations, existing evidence suggests, are not just passive markers of systemic inflammation. They function as highly sensitive physiological indicators, closely tracking how the host’s condition progresses from a localized respiratory infection to systemic multi-organ dysfunction (22–24).

Across large patient cohorts, longitudinal studies have repeatedly found that total cholesterol (TC) and low-density lipoprotein cholesterol (LDL-C) levels drop significantly as the disease worsens (13, 25, 26). What stands out is that differences in lipid profiles start to emerge as early as 48–72 hours after enrollment. This is most evident when comparing patients who later develop ARDS to those who do not. When admitted to the hospital, severe cases almost always have much lower TC and LDL-C levels than patients with mild disease (27).

Looking at these observed lipid changes in severe viral pneumonia, several clinical studies have found a clear connection between lower LDL-C levels at admission and both more severe disease and worse clinical outcomes for affected patients (26, 28). This systemic hypocholesterolemia is not just a side effect of malnutrition or liver dysfunction. Rather, it stems from the significant “cholesterol drain” needed to build viral envelopes and form specialized “lipid rafts”—structures that are essential for viral entry—and available experimental evidence supports this mechanism. Triglyceride (TG) concentrations, too, often change dynamically over the course of viral pneumonia. Changes in TG metabolism have been linked to disease progression, but the exact timing of these changes and the mechanisms behind them still need more research (20, 29, 30).

### 2.2 High-density lipoprotein and apolipoprotein A-I: the failure of the “immunometabolic shield” and demographic resilience

Among all circulating lipids and proteins, ApoA-I and its associated HDL particles have emerged as the most robust predictors of 28-day mortality in the analyzed literature set (31, 32). HDL particles traditionally serve as the body’s “immunometabolic shield,” capable of neutralizing lipopolysaccharides (LPS) and downregulating the expression of endothelial adhesion molecules (33, 34). ROC curve analysis in critically ill patient cohorts has validated the strong prognostic performance of serum ApoA−I for adverse clinical outcomes. Across multiple studies in severe systemic inflammatory disorders, ApoA−I shows high discriminative value with Area Under the Curve (AUC) values in the range of 0.88 to 0.93, supporting its utility as an early predictive marker for clinical deterioration (35, 36). Notably, low serum ApoA−I levels at admission are closely associated with prolonged hospital stay, increased intensity of care, and poor clinical prognosis in critically ill populations (12, 31, 37).The sharp reduction in ApoA−I observed during severe infection is closely linked to inflammatory activation. Inflammatory mediators are known to modulate hepatic production and peripheral clearance of ApoA−I−containing lipoproteins, though detailed regulatory pathways remain to be fully defined in the setting of viral pneumonia (38).

However, A full understanding of HDL dysfunction requires more than a simple look into quantitative changes in circulating levels. It is imperative to resolve the qualitative and functional transformation of HDL particles during severe viral infection. Increasing evidence suggests that HDL undergoes dramatic pathological remodeling under viral assault - a process that is now becoming recognized as “proteomic switching” (39–41).

High-resolution mass spectrometry (LC-MS/MS) is used to show that, in acute phase inflammation, the anti-inflammatory protein ApoA-I is physically displaced from the HDL particle by Serum Amyloid A (SAA) and Complement C3. Accumulating data show that the relative abundance of SAA in the HDL fraction increases significantly in severe viral pneumonia, and this is accompanied by a corresponding reduction in ApoA-I content (42, 43). This converts “acute-phase HDL” (ap-HDL) to lose its canonical anti-inflammatory function, most notably the ability to promote reverse cholesterol transport (RCT) from pulmonary macrophages (44).

Instead, SAA-enriched ap-HDL has strong pro-inflammatory and pro-oxidative effects in the pulmonary microenvironment. While the exact receptor-mediated signaling pathways still need to be validated, there is existing evidence to confirm the role of ap-HDL in the activation of endothelial cells and enhancing inflammatory responses in virus-induced lung injury (45, 46). This functional change of HDL worsens the leakiness of the blood vessels, which is one of the hallmarks of viral associated ARDS.

Furthermore, HDL particles are extensively modified by myeloperoxidase (MPO) in critically ill patients, and these particles become carbonylated and nitrated at residual ApoA-I molecules. This oxidized HDL (ox-HDL) loses its native capacity to inhibit vascular cell adhesion molecule-1 (VCAM-1) expression on endothelial cells (47, 48). In turn, ox-HDL acts as a “pro-inflammatory sword” promoting the progression of local lung injury leading to systemic inflammatory response syndrome (SIRS) through lasting activation of endothelial dysfunction and enhanced systemic inflammation (45, 49).

This profound metabolic reprogramming of HDL is not uniform across all individuals. Host responses to severe viral pneumonia are highly heterogeneous and are strongly related to baseline metabolic health. Recent studies have offered important insights into the concepts of “Lipid Frailty” and sex-specific dimorphism (50–52).

The concept of lipid-related metabolic vulnerability has been used to describe older adults (aged >65), who exhibit a reduced ability to restore lipid homeostasis after acute viral infection. In these individuals, a persistent decrease in HDL-C and an increase in sphingomyelin concentrations beyond the acute phase are associated with worse long-term clinical outcomes and are meaningful predictive indicators for metabolic disturbances associated with Long COVID (53–55).

Moreover, the “Proteomic Switching” of HDL exhibits a distinct gender bias. Male patients consistently have a more pronounced decrease in protective apolipoprotein A-1 and a much higher elevation of pro-inflammatory sphingolipids than female patients (50, 51). This metabolic gap may explain the observed higher mortality rate in males, whose lipidomic profile is shifted towards a pro-oxidative and pro-thrombotic state earlier in the disease course.

### 2.3 Phospholipid remodeling and alveolar-capillary barrier failure: the LPC/PC axis

The integrity of the alveolar-capillary membrane is critically dependent upon the exact nature of the compositional profile of its constituent phospholipids. Emerging clinical metabolomic information suggests that the most specific molecular signature of lethal viral pneumonia is not a generalized reduction in lipid content, but rather an extensive and selective depletion of lysophosphatidylcholines (LPC) (22, 56, 57). This depletion is particularly significant because LPC species are primarily transported by ApoA-I-containing HDL particles. Thus, the collapse of systemic LPC pools represents not only a failure in inflammation resolution but also a key biochemical mechanism underpinning the prognostic value of ApoA-I decline. High resolution LC-MS/MS profiling further shows that circulating levels of LPC are significantly lower in patients who develop ARDS than in patients presenting with milder phenotypes of clinical disease (56, 58, 59).

This depletion is particularly high in LPC subspecies enriched in polyunsaturated fatty acids (PUFAs) such as LPC 18:2, LPC 20:4 and LPC 22:6. These lipids of the PUFA category are recognized precursors in the biosynthesis of Specialized Pro-Resolving Mediators (SPMs), such as resolvins and protectins. A collapse in systemic LPC pools may therefore compromise the host’s capacity to generate these pro-resolving lipid mediators, potentially leading to a state of “resolution failure” in which inflammatory cascades cannot be properly terminated. Such a defect is assumed to be involved in the development of persistent, non-resolving lung injury seen in patients with fatal viral pneumonia (60–62).

Moreover, this phospholipid remodeling is closely associated with enhanced activation of secretory phospholipase A2 (sPLA2-IIA). In critically ill patients, elevated sPLA2 activity is associated with reduced circulating LPC levels. This inverse association, while initially counterintuitive given sPLA2-IIA’s enzymatic function, likely reflects a combination of local compartmentalization of enzyme activity, alterations in other LPC-metabolizing enzymes (LCAT and LPCAT), and substrate depletion under sustained inflammatory conditions. The precise mechanisms in human viral pneumonia remain to be fully elucidated. Nevertheless, an elevated serum PC/LPC ratio is strongly correlated with impaired pulmonary surfactant function, increased surfactant protein-D (SP-D) leakage, and has become a valuable prognostic tool to identify patients with increased risk of refractory hypoxemia (63–66).

### 2.4 Sphingolipid dysregulation: ceramides as death signals and thrombo-inflammatory scaffolds

In stark contrast to the “lipid crash” seen in cholesterol and phospholipids, the sphingolipidome exhibits a paradoxical and deadly elevation. Ceramides (Cer) and sphingomyelin (SM) are emerging as strong pro-apoptotic mediators in the late-phase pathology of viral pneumonia (67, 68).

Quantitative profiling reveals a unique “lethal signature” of long-chain ceramides involving Cer d18:1/24:0 and Cer d18:1/16:0 that are highly increased in the serum and bronchoalveolar lavage fluid (BALF) of patients with diffuse alveolar damage (DAD). These lipids act as second messengers involved in the extrinsic apoptosis pathway of alveolar type II (AECII) cells by Fas-mediated signaling (69–71). The consequent massive epithelial shedding is not only responsible for the impairment of gas exchange, but also for the formation of hyaline membranes.

Beyond apoptosis, the build-up of ceramides on the endothelial surface forms a key nexus to the coagulopathy that is seen in severe cases. Circulating levels of ceramide are highly correlated with markers of immunothrombosis, including D-dimer and PAI-1. Mechanistically, ceramide-enriched platforms generate a pro-coagulant surface that facilitates thrombotic pathway activation directly connecting metabolic failure in the host to microvascular occlusion and multiorgan dysfunction that defines the disease (72, 73).

### 2.5 Precision medicine: machine learning and metabolic endotyping

High-dimensional lipidomic profiling has provided new opportunities for risk stratification and individual therapeutic intervention in severe pneumonia. The conversion of lipid signatures into clinical decision-making processes is one of the key trajectories for precision medicine (74–76).

Utilizing advanced machine learning algorithms, such as Random Forest and XGBoost, researchers have developed predictive models that outperform traditional clinical scoring systems for risk stratification in severe pneumonia. These models have been shown to have good prognostic power for mortality risk assessment, especially in the first 24 hours of hospital admission (77–79).

More critically, lipidomics enables pneumonia patients to be sub-phenotyped into discrete “Metabolic Endotypes.” The Hyper-inflammatory Endotype (characterized by low LPC and high Cer) requires aggressive immunomodulation and potentially specialized pro-resolving lipid emulsions, whereas the Hypo-metabolic Endotype (characterized by global lipid depletion) may benefit from early nutritional lipid support. Identification of these endotypes at admission represents a prerequisite for the implementation of personalized immunometabolic therapy in contemporary intensive care units (22, 24, 80).

## 3 Molecular mechanisms of lipidomic reprogramming

### 3.1 The viral hijacking of host transcription: SREBP-mediated lipid synthesis and replication organelles

The successful replication of respiratory viruses in the nutrient-starved environment of the infected lung requires the rapid acquisition of specialized lipid species. Notably, although SARS-CoV-2 (+ssRNA) and IAV (−ssRNA) differ in replication compartment (ERGIC-derived DMVs vs. nuclear replication) and budding site (intracellular vs. plasma membrane lipid rafts), both actively reprogram host transcription via the Sterol Regulatory Element-Binding Protein (SREBP) axis—with SREBP-2 driving cholesterol-dependent DMV biogenesis in SARS-CoV-2 and SREBP-1 facilitating fatty acid-dependent glycoprotein palmitoylation in IAV—turning the alveolar epithelium into a high-output “lipid factory” (81–83).

**Figure 1:**
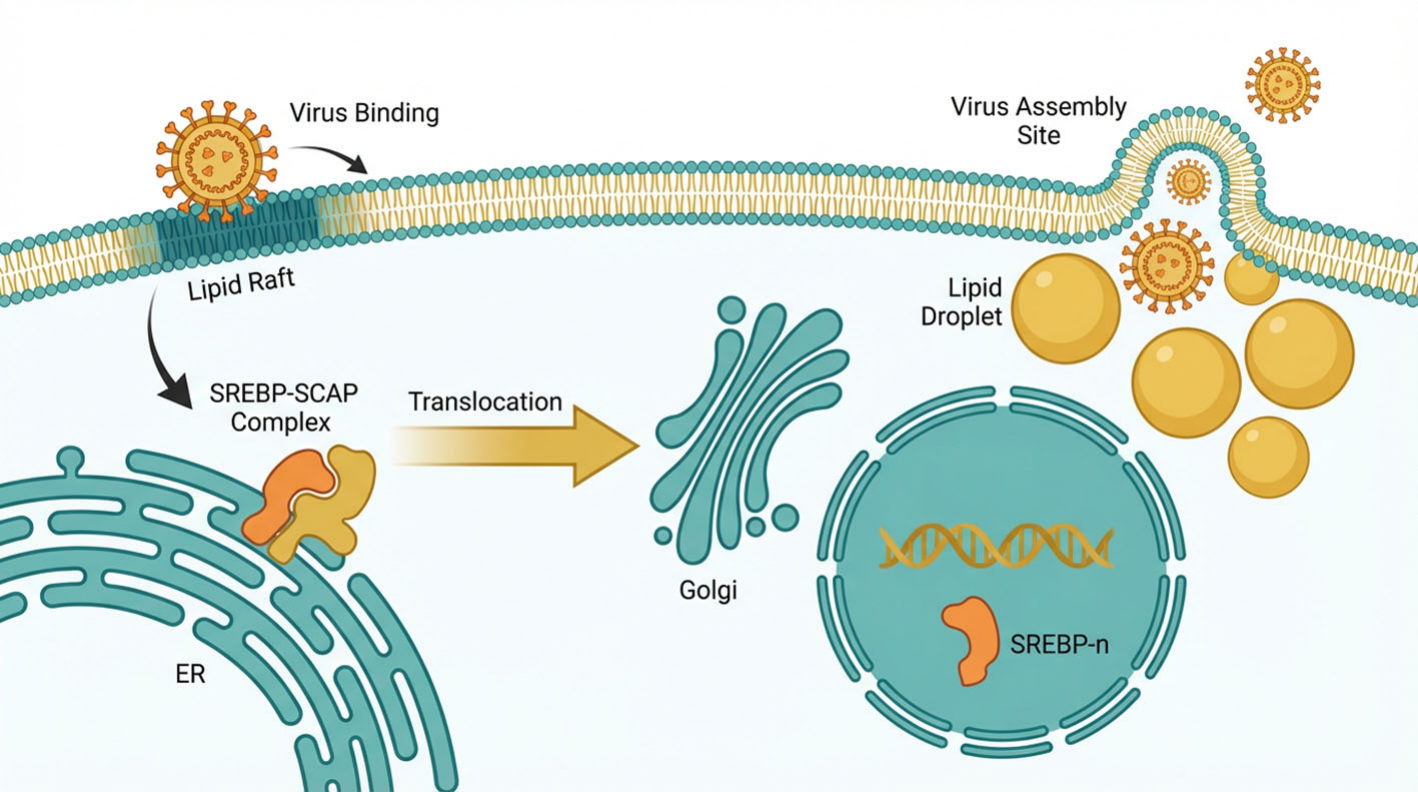
Viral Subversion of Host Lipid Metabolism.

Under physiological conditions, SREBPs, namely SREBP-1 and SREBP-2 that control fatty acid synthesis and cholesterol synthesis, respectively, are sequestered within the endoplasmic reticulum (ER) together with the chaperone protein SCAP. Upon infection, viral accessory proteins, such as the SARS-CoV-2 ORF3a protein or the IAV hemagglutinin protein, cause severe ER stress and perturbations in calcium signaling and consequently the proteolytic cleavage of SREBPs and subsequent nuclear translocation (81, 84). Once in the nucleus, SREBP-1 potently up-regulates the transcription of the entire *de novo* lipogenic program, especially of Fatty Acid Synthase (FASN) and Acetyl-CoA Carboxylase (ACC). This up-regulation causes an ample metabolic flow to the production of palmitate and long-chain saturated fatty acids, which are crucial for the assembly of double-membrane vesicles (DMVs). These DMVs serve as “viral replication organelles” that shield viral RNA from host innate immune sensors, such as MDA5 and RIG-I, thereby allowing for unchecked viral proliferation within the alveolar space (83, 85, 86) (**Figure 1**).

This transcriptional hijacking further manifests in a large accumulation of lipid droplets (LDs) in infected alveolar type II (AT II) epithelial cells. Far from being inert energy-storage organelles, LDs are recycled as important physical scaffolds for the assembly and budding of viruses. High-resolution imaging and proteomic profiling shows that virus nucleocapsid (N) proteins preferentially target the LD surface, recruiting the host protein adipose differentiation-related protein (ADRP) in order to stabilize these structures against lipophagy (87, 88). Mechanistically, the virus utilizes LD-derived triacylglycerols (TAGs) to fuel the immense energetic demands of genome replication, while simultaneously utilizing the high-curvature LD surface to facilitate the assembly of the viral envelope. Clinical evidence suggests that pharmacological inhibition of the SREBP–SCAP axis or pharmacological inhibition of diacylglycerol acyltransferase 1 (DGAT1) to stop the formation of the LDs can significantly lower viral titers, demonstrating that this metabolic hijacking is an obligate step in the pathogenesis of viral pneumonia (84, 87, 89).

### 3.2 The CH25H/25-OHC axis: a molecular pivot between defense and hyper-inflammation

The reviewed literature uncovers a second mechanism: a sophisticated yet pathological modulation of the Cholesterol 25-Hydroxylase (CH25H) pathway. This axis is part of a central interferon-stimulated gene (ISG) response that evolved as a host defense mechanism, but is often hijacked by virulent respiratory viruses to become a cause of tissue destruction (90–92).

During the early “viremic” phase of infection, type I interferons (IFN-alpha/beta) upregulate expression of CH25H in alveolar macrophages and resident epithelial cells in a robust manner. The resulting product, 25-hydroxycholesterol (25-OH-C), acts as a powerful antiviral oxysterol, by inducing the changes in the cholesterol composition of the host cell plasma membrane that result in the inhibition of the membrane fusion needed for entry of the virus: in the case of the current study, the fusion mediated by the spike protein of the 2019 coronavirus (SARS-CoV-2) and the hemagglutinin-mediated entry of influenza viruses (90, 91, 93). In addition, 25-OHC functions as a natural ligand for the liver X receptors (LXRs), inducing cholesterol efflux and effectively “starving” the virus of the sterols needed as a component of its lipid raft-dependent entry (94).

However, as the infection enters the “hyper-inflammatory” phase, the CH25H/25-OH axis undergoes a catastrophic functional pivot. Elevated concentrations of 25-OHC found in bronchoalveolar lavage fluid (BALF) are a potent chemoattractant for monocytes and neutrophils mediated through the GPR183 (EBI2) receptor (95, 96). Increased levels of certain cholesterol metabolites are directly related to the extent of the cytokine storm. Mechanistic studies have shown that activation of the NLRP3 inflammasome in pulmonary macrophages contributes to the increased production of IL-1β and IL-6 that worsen vascular leak and epithelial denudation, hallmarks of DAD (97–99). This molecular switch explains how a defensive lipid response may be transformed into a cause of lethal lung damage and identifies cholesterol metabolite-mediated inflammatory pathways as attractive targets for immunometabolic intervention in severe viral pneumonia.

### 3.3 Ferroptosis and lipid peroxidation: the biochemical executioner of alveolar integrity

One major mechanistic understanding is the discovery of ferroptosis, a type of iron-dependent, non-apoptotic regulated cell death marked by the catastrophic accumulation of lipid peroxides, as a major cause of diffuse alveolar damage (DAD) in severe viral pneumonia (100, 101). Unlike apoptosis, which is immunologically silent, ferroptosis results in an enormous release of damage-associated molecular patterns (DAMPs), thus precipitating the systemic “cytokine storm” (102, 103).

The molecular initiation of ferroptosis in the infected lung revolves around the inhibition of the System Xc^-^/glutathione (GSH)/GPX4 axis. Viral replication presents a tremendous oxidative load to the host cell, which results in the rapid depletion of intracellular GSH. Specifically, viral proteins have been found to down-regulate SLC7A11, the catalytic subunit of the cystine/glutamate antiporter, thereby effectively starving the cell of the precursor needed for GSH synthesis (104, 105). This depletion leads to the inactivation of glutathione peroxidase 4 (GPX4), the “master guardian” of the lipidome and renders it catalytically inactive. In the absence of functional GPX4, the cell loses its ability to neutralize highly reactive lipid hydroperoxides (LOOH), especially those that are located inside the phospholipids that contain polyunsaturated fatty acids (PUFAs) in the plasma and mitochondrial membranes (106, 107).

The biochemical “executioner” in this process is the Fenton reaction in which excess labile intracellular iron (often from ferritin complexes that have been hijacked) catalyzes the conversion of lipid hydroperoxides into the lethal alkoxyl (LO•) and peroxyl (LOO•) radicals (108, 109). Emerging evidence reveals that viruses degrade ferritin primarily through NCOA4-mediated ferritinophagy, a selective autophagic process in which the cargo receptor NCOA4 binds to ferritin and delivers it to autophagosomes for lysosomal degradation, thereby releasing iron stores (110). In influenza A virus (IAV) infection, the viral hemagglutinin (HA) protein interacts with autophagic receptors NCOA4 and TAX1BP1, facilitating the formation of ferritin-NCOA4 condensates and potently inducing ferritinophagy. HA-induced ferritinophagy leads to cellular lipid peroxidation and impairs MAVS-mediated antiviral immunity, thereby promoting viral replication (111). In SARS-CoV-2 infection, virus-induced hyperinflammation—particularly elevated interleukin-6 (IL-6)—promotes hyperferritinemia, which in turn triggers NCOA4-mediated ferritinophagy and iron release, thereby exacerbating ferroptosis and tissue damage (112). Collectively, these findings identify IAV and SARS-CoV-2 as key viruses capable of promoting ferritin breakdown via NCOA4-mediated ferritinophagy.

In parallel with ferritinophagy, mechanistic analysis identifies specific viruses as doing so due to up-regulation of acyl-CoA synthetase long-chain family member 4 (ACSL4). This enzyme selectively enriches the membrane with arachidonic acid (AA) and adrenic acid (AdA) containing phosphatidylethanolamines (PE-AA/AdA) that are the main substrate for lipid peroxidation (113, 114). The subsequent oxidative breakdown of these membrane lipids precipitates the formation of pores in cellular membranes, a catastrophic loss in membrane potential, and eventually necrotic rupture of the alveolar epithelial and endothelial barriers. Clinical abstracts show that administration of ferrostatin-1 or iron chelators can significantly reduce lung injury and pro-inflammatory cytokine production in lethal viral models, identifying the ferroptotic pathway as a critical therapeutic window (101, 115).

**Figure 2:**
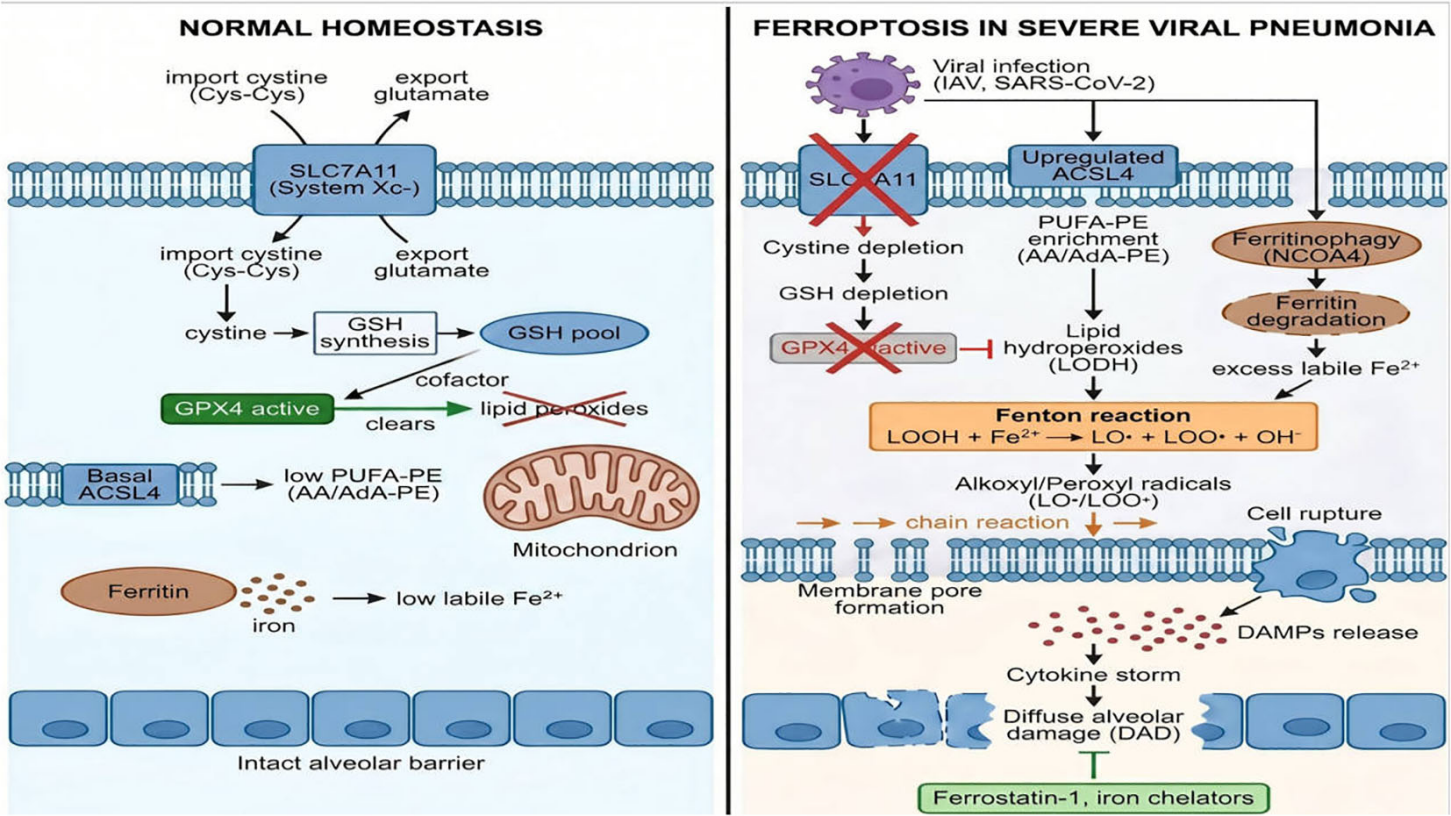
Ferroptosis pathway in alveolar epithelial cells during severe viral pneumonia.

To summarize, the ferroptotic pathway from viral infection to alveolar barrier disruption can be delineated as the following sequential steps (**Figure 2**):

Step 1: Viral infection → downregulation of SLC7A11 → depletion of cystine and GSH.
Step 2: Inactivation of GPX4 → loss of lipid peroxide scavenging capacity.
Step 3: ACSL4-mediated enrichment of membrane PUFA-PE (AA/AdA) as peroxidation substrates.
Step 4: Accumulation of labile Fe^2+^ (from ferritin breakdown) → Fenton reaction converts LOOH to lethal alkoxyl/peroxyl radicals.
Step 5: Lipid peroxidation chain reaction → membrane pore formation → necrotic rupture of alveolar epithelial/endothelial barriers.
Step 6: Release of DAMPs → amplification of cytokine storm → DAD.

### 3.4 The interplay between lipid peroxidation and pro-inflammatory signaling: the 4-HNE bridge

Lipid peroxidation has pathological consequences that go beyond the rupture of membranes because the products of lipid peroxidation act as potent bioactive signaling mediators that can amplify pulmonary inflammatory responses. Among these metabolites, 4-hydroxynonenal (4-HNE), a reactive α,β-unsaturated aldehyde generated during the breakdown of ω-6 polyunsaturated fatty acids (PUFAs), is a major one (116, 117).

Elevated concentrations of 4-HNE protein adducts have been found in lung tissues of patients with severe viral pneumonia. 4-HNE acts as a molecular bridge between metabolic perturbation and immunological activation, and its impact appears to be context-dependent (e.g. dose- and cell type-specific) (118, 119). Firstly, 4-HNE is able to directly modify the IKK complex, although investigations have reported conflicting effects on NF-κB activation. While some research shows that 4-HNE suppresses NF-κB signaling through impaired degradation of IκB-α and suppression of TNF-α production, one study in an atherosclerosis model showed increased NF-κB activity and excessive production of TNF-α, IL-6 and IL-8 (120–123). Secondly, 4-HNE-modified proteins can regulate Toll-like receptor 4 (TLR4) mediated immune responses. Evidence indicates that 4-HNE can either inhibit TLR4 activation or enhance TLR4/NF-κB signaling, depending on the cellular milieu (124, 125).

Moreover, 4-HNE has been reported to inhibit the antioxidant response mediated by the nuclear factor erythroid 2-related factor 2 (Nrf2) during the late stages of the infection. Although Nrf2 first tries to reduce oxidative stress, the deposition of 4-HNE causes carbonylation of Keap1 and proteasome degradation of Nrf2, which locks the alveolar environment in a state of chronic oxidative susceptibility (126, 127). This ongoing oxidative-inflammatory feedback cycle is a feature of the transition from acute pneumonia to the fibroproliferative phase of ARDS. Identification of intervention points within this 4-HNE/NF-κB/Nrf2 nexus is imperative for the development of “metabolic stabilizers” able to protect the lung from collateral damage caused by the host’s own inflammatory response (128, 129).

### 3.5 The sphingolipid rheostat: ceramide-mediated mitotoxicity and alveolar apoptosis

The lethal course of viral pneumonia is intrinsically bound up in the loss of the “sphingolipid rheostat” — the homeostatic balance between pro-apoptotic ceramides and pro-survival sphingosine-1-phosphate (S1P). Systematic analysis has shown that virulent respiratory viruses direct a massive metabolic reconfiguration towards ceramide accumulation via two synergistic pathways: the stress activated activation of acid sphingomyelinase (aSMase), and the transcriptional upregulation of serine palmitoyltransferase (SPT), rate-limiting enzyme in *de novo* synthesis of sphingolipids (130–133).

The main pathological manifestation of this ceramide onslaught is the disastrous failure of mitochondrial bioenergetics in AECII cells. At high micromolar concentrations, ceramide molecules have a unique biophysical property in that they self-assemble into large protein-independent channels in the mitochondrial outer membrane (MOM) (134). These ceramide channels are responsible for the non-selective efflux of pro-apoptotic factors, most notably cytochrome c, from the intermembrane space to the cytosol. In clinical lung biopsies from patients with diffuse alveolar damage (DAD), elevated levels of long-chain ceramides are linked to increased mitochondrial reactive oxygen species (mtROS) production and subsequent activation of the caspase-9/3 proteolytic cascade (70, 135, 136). This mitochondrial “suicide program” produces the extensive denudation of the alveolar epithelium, in a fundamental way compromising gas exchange, precipitating the transition to refractory hypoxemia.

### 3.6 S1P signaling deficit and the breakdown of the endothelial “molecular glue”

While ceramides carry out the epithelial cell death program, the concurrent depletion of Sphingosine-1-Phosphate (S1P) acts as the major agent of vascular permeability and pulmonary edema. S1P, which signals primarily via the S1PR1, acts as a “molecular glue” that maintains endothelial barrier integrity by stabilizing the VE-cadherin complex at adherens junctions (137, 138).

Mechanistic studies within the data set indicate that viruses use non-structural proteins (NSPs) to specifically target and inhibit sphingosine kinase 1 (SPHK1), and not all high-pathogenicity viruses do so in the same uniform way. Sphingosine kinase 1 is an enzyme involved in converting the pro-apoptotic sphingosine to protective S1P, and its inhibition thus leads to reduced S1P production (139, 140). When circulating levels of S1P drop below a critical level, the S1PR1-mediated signaling axis collapses, leading to breakdown of the pulmonary microvasculature. This “metabolic breach” allows a tremendous amount of protein-rich fluid to enter the alveolar space — the hallmark of non-cardiogenic pulmonary edema in ARDS. Experimental evidence has shown that therapy using S1PR1 agonists or activators of SPHK1 can restore the endothelial barrier and substantially reduce the lung injury score, therefore further supporting the ceramide/S1P imbalance as a fundamental lethal mechanism in viral pneumonia (140–142).

### 3.7 Lipid-protein interactomics: modulating innate immune sensors and viral evasion

The molecular complexity of dysregulated lipidomes is further complicated by direct interactions between particular lipid species and host innate immune sensors. In addition to acting as structural components or sources of metabolic energy, lipids act as “molecular switches” to fine-tune the sensitivity of antiviral responses (143, 144).

A representative example is the role of membrane cholesterol in the modulation of the STING (Stimulator of Interferon Genes) pathway. In the late stages of infection, virus-induced cholesterol depletion activates the STING pathway because low cholesterol levels promote STING-dependent Type I Interferon (IFN) induction by promoting its translocation from the endoplasmic reticulum (ER) membrane to the Golgi complex — a necessary step for the induction of Type I IFNs. This lipid-mediated modulation implies that viruses may use cholesterol to turn down STING signaling and enable viral escape from host surveillance despite increasing viral load (145, 146). Concurrently, pathological accumulation of 25-hydroxycholesterol (25-OHC) in alveolar macrophages provides a second pro-inflammatory impulse through direct activation of the NLRP3 inflammasome scaffold, thereby causing unregulated maturation and release of IL-1β and IL-18 (97, 98). This synergy between lipid structural changes and the amplification of immune signaling explains the “uncoupling” of the inflammatory response and viral clearance wherein the host lipidome becomes a weapon that is used by the pathogen to destroy the very immune defenses that were designed to contain them (98, 144).

## 4 Systemic regulation: the gut-lipid-lung axis and metainflammation

### 4.1 The gut-lipid-lung axis: microbial orchestration of pulmonary immunity

One of the most profound changes in respiratory immunometabolism is the understanding that the lipid environment of the lung is not an isolated compartment, but may be continually recalibrated by the gastrointestinal tract—a proposed two-way communication framework called the Gut-Lipid-Lung Axis, expanding on the well-established concept of the Gut-Lung Axis (147, 148). Analyses suggest that gut dysbiosis during viral pneumonia is not simply a secondary complication of antibiotic use or systemic inflammation that causes lipidomic dysfunction in the respiratory tract, although this hypothesis has been supported by a range of plausible mechanisms that can link the status of gut microbial communities to the lung’s lipid-immune environment (149).

**Figure 3:**
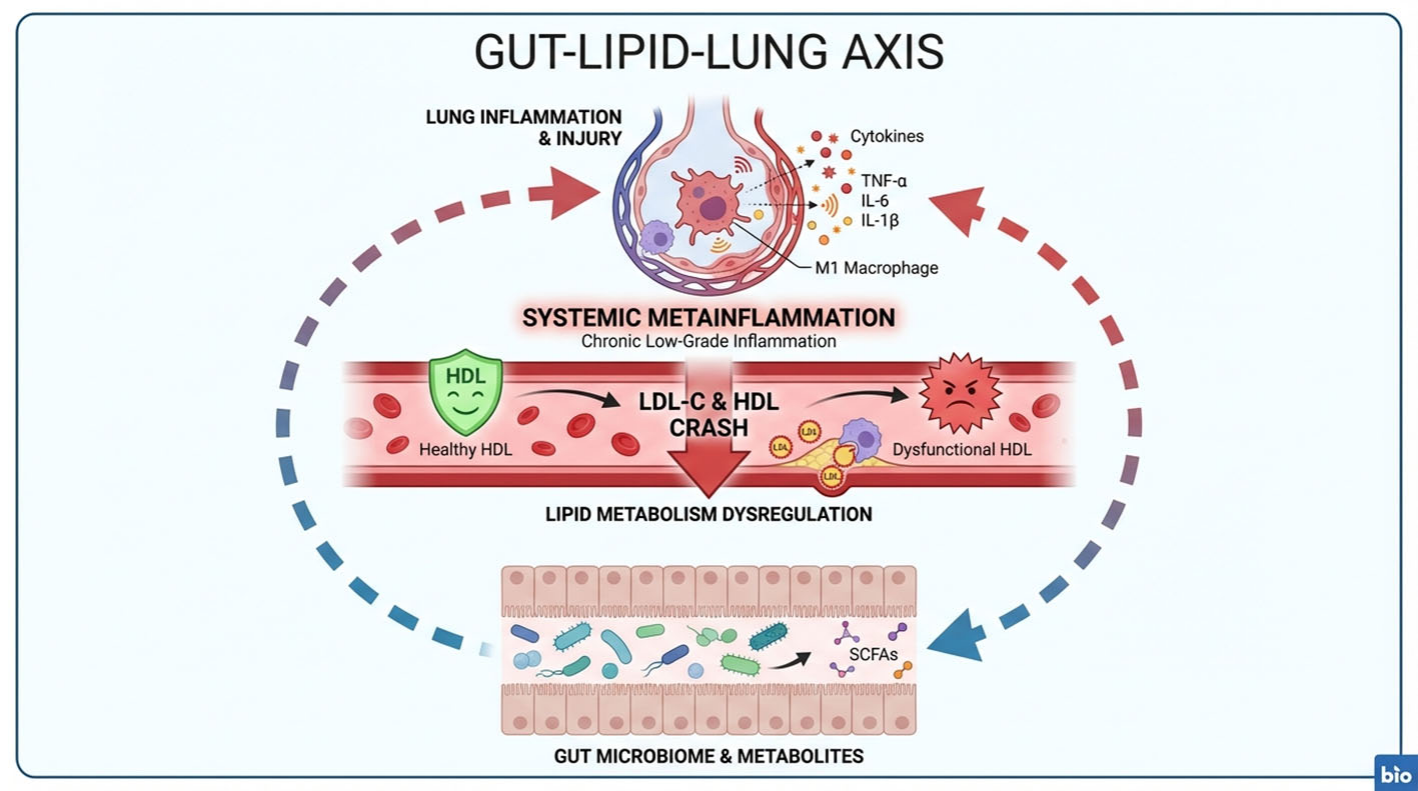
The gut-lipid-lung axis and systemic metainflammation.

The molecular messengers of this axis are in the main short-chain fatty acids (SCFAs), in particular acetate, propionate and butyrate, produced by the bacterial fermentation of dietary fibers. SCFAs are high-affinity ligands of G-protein-coupled receptors (GPR41 and GPR43) expressed on gastrointestinal enteroendocrine cells and on immune progenitors (150, 151). Mechanistically, SCFAs are absorbed into the portal circulation and regulate the liver’s lipid synthesis, particularly shifting the liver to produce unsaturated phosphatidylcholines (PCs) and inhibiting the systemic release of pro-inflammatory ceramides. In the setting of viral pneumonia, a depleted SCFA pool (due to virus-induced gut barrier breakdown) causes a systemic surge in “primed” neutrophils and a simultaneous decrease in the anti-inflammatory capacity of alveolar macrophages (AMs). Clinical data from the cited studies correlate a high butyrate:ceramide ratio in the serum with a significantly lower risk of progressing to secondary bacterial superinfection, highlighting the gut as a distant “metabolic rheostat” for pulmonary defence (152–154) (**Figure 3**).

### 4.2 Alveolar macrophage polarization: harvesting circulating lipids for immune effector function

The functional plasticity of alveolar macrophages (AMs), the main sentinels of the lung, is strictly controlled by their ability to acquire and metabolize lipids from the systemic circulation and the alveolar surfactant pool (155, 156).

During the progression of the viral infection, AMs undergo metabolic reprogramming, which determines the polarization state of these cells. Analysis identifies a pivotal role for the liver X receptor (LXR) signaling pathway in this process. When AMs sense an increase in oxysterols (such as 27-hydroxycholesterol) from the systemic circulation, LXR activation promotes the expression of cholesterol efflux transporters such as ABCA1 and ABCG1. This “cholesterol unloading” is important for maintaining the M2-like, pro-resolving phenotype of the macrophage. Conversely, in the case of severe pneumonia, a systemic “lipid crash” results in the AMs being deprived of these regulatory ligands, thus pushing them into a cholesterol-clogged, M1-like state (157–160). These lipid-congested macrophages exhibit a hyperresponsive NLRP3 inflammasome as well as decreased role for efferocytosis (i.e., the cleaning up of apoptotic alveolar epithelial type II cells). The failure of efferocytosis results in secondary necrosis in the alveoli, further increasing the amount of metabolic debris and continuing the cycle of tissue destruction. Consequently, the metabolic state of the AM can be considered as the gateway to connect the fluctuation of lipidomics in the entire body to the local pathology in the lungs (161–163).

### 4.3 Metainflammation: the lethal synergy of obesity and viral pneumonia

A large body of the literature reviewed is centered around the clinical paradox of metainflammation, i.e., the chronic, low-grade inflammatory condition associated with obesity and metabolic syndrome, as a predisposing condition to lethal viral pneumonia (164–166).

In obese subjects, this enlarged adipose mass acts as a major store for pro-inflammatories (adipokines, e.g. leptin) and saturated fatty acids. Systemic lipid perturbations associated with obesity have been proposed to precondition the pulmonary microvasculature and therefore may contribute to increased endothelial responsiveness before viral challenge. Upon infection, these patients can present an excessive lipidomic shift; it has been postulated that this shift is associated with an imbalance between protective apolipoprotein A-1 and pro-inflammatory serum amyloid A (SAA)-HDL, as well as modifications of the basal expression of long-chain acyl-CoA synthetase 4, considered an important enzyme in the induction of ferroptosis (167–169). This metabolic pre-priming highlights the idea that obesity is not just a comorbidity but a fundamental cause of the hyper-inflammatory phenotype. Evidence suggests that mortality risk in these patients is not necessarily just proportional to viral load, but rather is the result of an abnormally vigorous lipid-cytokine feedback loop that leads to earlier and more severe diffuse alveolar damage (DAD) (164, 170, 171).

### 4.4 Bioactive lipid mediators as rheostats of the “cytokine storm”

The systemic inflammatory response seen in viral pneumonia is not merely triggered by viral pathogen-associated molecular patterns (PAMPs), but is sustained and amplified by a family of bioactive lipid mediators that serve as the “second messengers” of the innate immune system. Analyses reveal that the development of a localized “cytokine storm” from a controlled inflammatory state is preceded by a systemic alteration in the eicosanoid profile (172–174).

In severe manifestations, the massive liberation of arachidonic acid (AA) from the injured cell membranes becomes a substrate for the synthesis of pro-inflammatory prostanoids. Specifically, a systemic rise in prostaglandin E2 (PGE2) and leukotriene B4 (LTB4) is a strong chemotactic signal, mobilizing the alveolar deposition of neutrophils that express CXCR2. In contrast to most bacterial pneumonias, virus-induced acute respiratory distress syndrome (ARDS) exhibits a “trapping” phenomenon, where these recruited neutrophils, due to increased local levels of lysophosphatidylcholine (LPC 16:0) and platelet-activating factor (PAF), have delayed apoptosis. This lipid-mediated persistence of neutrophils leads to excessive release of neutrophil extracellular traps (NETs), which go on to entangle virions and fibrin, thus giving rise to the “gel-like” hyaline membranes, which impair gas exchange (175–177). Beyond the global lipidomic remodeling discussed above, the balance between specific eicosanoid mediators—pro-inflammatory leukotriene B4 (LTB4) and pro-resolving lipoxin A4 (LXA4)—has been implicated in the pathogenesis of persistent neutrophilic inflammation. Studies in COPD and bronchiectasis have documented an elevated LTB4/LXA4 ratio in airway samples, reflecting a shift toward a pro-inflammatory eicosanoid profile (174, 175). Whether this lipid mediator imbalance contributes to the sustained neutrophilic infiltration and “neutrophilic congestion” observed in severe viral pneumonia warrants further investigation (178, 179).

### 4.5 Adipose-lung crosstalk: the role of adipokines and ectopic lipid deposition

The systemic control of pulmonary inflammation is further complicated by the endocrine function of adipose tissue. In the context of metainflammatory disease, the “Adipose-Lung Axis” appears to be a major driver of illness severity. Analysis shows that leptin, an adipokine that is raised during obesity, has a direct metabolic programming effect on AECII (180, 181).

Elevated systemic concentrations of leptin have been linked to a putative “pro-glycolytic” shift in AECII cells, a modification that may have been initially necessary to meet the high energetic demands of surfactant synthesis, but which may also cause mitochondrial stress and render AECII cells susceptible to ceramide-induced apoptosis. In addition, there is evidence of ectopic lipid deposition in the pulmonary parenchyma of patients with the metabolic syndrome. These “lipid-laden” interstitial cells are associated with the secretion of IL-6 and MCP-1, which may further promote the inflammatory environment of the lung (182, 183). Such changes create a “pre-heated” pulmonary milieu where exposure to a moderate viral attack can trigger an exponential inflammatory cascade. Clinical reports show that the extent of visceral adiposity, as measured by mid-axial computed tomography, is more predictive of the “Lipid-Cytokine Feedback Loop” than is body mass index alone, providing yet more evidence for the need for body composition-aware metabolic phenotyping in the intensive care environment (184–186).

## 5 Therapeutic implications and metabolic intervention

### 5.1 Targeted modulation of lipogenesis: the SREBP-SCAP axis and FASN inhibition

The realization that the SREBP-1/2 signaling axis represents a key viral hijacking site has led to a shift towards the use of metabolic inhibitors as host-directed therapeutic (HDT) approaches to viral pneumonia. Evidence suggests that *de novo* lipogenesis can be efficiently inhibited pharmacologically and thereby deprive the virus of substrates for the biogenesis of the replication organelles (83, 187, 188).

Fatostatin, a small molecule antagonist of the SCAP-SREBP complex, is a major candidate in this class. By blocking ER-to-Golgi trafficking, Fatostatin has shown antiviral activity in the lungs with data demonstrating decreases in double membrane vesicle formation and inhibition of viral replication across multiple viral species (81, 189). Additionally, inhibitors of fatty acid synthase (FASN), such as orlistat and the more selective TVB-2640, have been shown to inhibit the palmitoylation of viral proteins – a process important for their functional membrane anchoring (190, 191).

Clinical abstracts suggest that although these inhibitors remain potent in the early viremic stage, their administration in late-stage acute respiratory distress syndrome (ARDS) requires careful titration to avoid interfering with the host’s endogenous membrane repair systems. Nonetheless, the incorporation of SREBP inhibitors into early intervention regimens represents a paradigm shift from conventional ‘one bug-one drug’ approaches to antiviral therapy toward a broad-spectrum metabolic stabilization approach with decreased susceptibility to resistance via viral mutation (83, 192, 193).

### 5.2 Immuno-lipidomics and specialized pro-resolving mediators: the omega-3 revolution

The failure of inflammatory resolution suggests that the supplemental administration of Specialized Pro-resolving Mediators (SPMs)—specifically Resolvins, Protectins, and Maresins—could provide a therapeutic bridge to terminate the “cytokine storm” (18, 194, 195).

Preclinical and mechanistic investigations indicate that Omega-3 fatty acid-enriched emulsions rich in EPA and DHA can elevate systemic levels of Resolvin E1 (RvE1) and Protectin D1 (PD1). These specialized pro-resolving mediators do not have the broad and immunosuppressive effects like corticosteroids. Rather, they actively promote non-phlogistic macrophage recruitment and facilitate the clearance of apoptotic neutrophils and cellular debris, thereby promoting the physiological resolution of inflammation (196–198).

The mechanistic basis for SPM-targeted nutritional support is robust, with evidence that SPMs augment the phagocytic capacity of resolution phase macrophages. Specifically, SPMs increase the host’s ability to kill microbes, stimulate macrophage uptake of apoptotic neutrophils and enhance overall microbial clearance. Furthermore, omega-3 supplementation—known to upregulate SPM synthesis—has been associated with improved white blood cell response in humans, strengthening the case for SPM-targeted nutritional interventions (199–201).

However, a critical caveat remains: the timing of Omega-3 administration is paramount. Clinical observations indicate that anti-inflammatory lipid pools become increasingly depleted in the course of acute respiratory distress syndrome (ARDS), with late-stage supplementation yielding diminished therapeutic benefit (202). Although the concept of a lipidomic metabolic window of opportunity prior to irreversible exhaustion of the phospholipid pool is biologically plausible, modern clinical research has not used prospective lipidomic profiling to guide the decision of when to start treatment or to help stratify patients based on baseline lipid status.

### 5.3 Restoring the “immunometabolic shield”: statins and PCSK9 inhibitors

Severe pneumonia is often linked with profound hypocholesterolemia and high-density lipoprotein (HDL) dysfunction, prompting controversial but evidence-based repurposing of cholesterol-modulating agents. While statins (HMG-CoA reductase inhibitors) are traditionally used in the management of cardiovascular disease, their pleiotropic effects, especially their ability to stabilize endothelial barriers, are of significant relevance to viral pulmonary injury (203–205).

Large-scale observational studies are inconsistent in terms of the efficacy of statins. A meta-analysis of 25 cohort studies with over 147,000 patients found that unadjusted analyses did not show a mortality benefit but that with adjustment for possible confounders there was a statistically significant reduction of mortality. Subgroup analyses further indicate that patients who present with high baseline C-reactive protein (CRP) and low HDL-C (which is suggestive of a hyperinflammatory endotype) have a significant survival advantage on atorvastatin (206). Mechanistically, statins block the prenylation of small GTPases like RhoA, thereby inhibiting endothelial contraction and subsequent vascular leak (207, 208).

Emerging evidence indicates that PCSK9 inhibitors, typified by evolocumab, possibly have special promise in the intensive care unit (ICU) setting. PCSK9 normally promotes degradation of the low-density lipoprotein receptor (LDLR) and the very low-density lipoprotein receptor (VLDLR) that are part of the removal of bacterial endotoxins and viral pathogen-associated molecular patterns (PAMPs) from the circulation. Inhibition of PCSK9 increases hepatic expression of these receptors, thereby enhancing the rapid clearance of pro-inflammatory lipid-protein complexes and may restore lipid homeostasis throughout the body (209–211). Early phase clinical studies are suggestive of the potential beneficial metabolic effects of PCSK9 inhibition during severe systemic disease with implications for organ dysfunction and survival outcomes (212).

### 5.4 Mitigation of oxidative membrane rupture: ferroptosis inhibitors and iron chelation

The devastating disruption of the alveolar-capillary barrier is triggered by iron-dependent lipid peroxidation. As a prominent therapeutic target, the ferroptotic cascade offers a unique opportunity to prevent the necrotic breakdown of pulmonary tissue at the peak of viral replication (101, 213).

Ferrostatin-1 (Fer-1) and its second-generation analogues are the most promising in this class of compound. Experimental studies using pre-clinical models of acute lung injury have shown that Fer-1 significantly reduces the pulmonary levels of 4-hydroxynonenal (4-HNE) and malondialdehyde (MDA), and successfully halts membrane degeneration (214–216). Such treatment was associated with improved oxygenation and a significant reduction in systemic release of damage-associated molecular patterns (DAMPs) such as high mobility group box-1 (HMGB1). In addition, chelators of iron such as deferoxamine (DFO) and the more lung-permeable deferiprone have been shown to be effective in sequestering the labile iron pool (LIP) which is the means of the Fenton reaction. Iron chelation therefore not only limits direct oxidative injury to AECII cells, but also blocks viral access to iron-sulfur clusters essential for the activity of the viral RNA-dependent RNA polymerase (RdRp) (217–219).

The main challenge in the path to clinical translation is the timing of intervention. As ferroptosis is a very rapid process that follows glutathione peroxidase 4 (GPX4) depletion, ferroptosis inhibitors could be used as early stabilizing agents in patients with increased risk. Combination approaches that simultaneously target ferroptosis pathways and other pathogenic mechanisms have the potential to offer synergistic protection against both viral pathogenesis and tissue injury in the host (220–222).

### 5.5 The sphingolipid rescue: targeting the ceramide/S1P axis

The restoration of the sphingolipid rheostat is a promising strategy for maintaining structural integrity of pulmonary microvasculature and preventing apoptotic shedding of alveolar epithelial cells (68, 223).

Current scholarly discourse puts a main focus on the repurposing of S1PR1 modulators, such as siponimod and fingolimod. These agents act as endothelial stabilizers by imitating the protective signaling by means of sphingosine-1-phosphate, thereby strengthening the VE-cadherin complexes and reducing vascular leakage. Consequently, S1PR1 modulators are considered promising candidates for the management of severe pneumonia with proposed benefits including reduction of pulmonary oedema and decreased dependence on mechanical respiratory support (224–226).

In addition to S1PR1 agonism, the inhibition of acid sphingomyelinase (aSMase) by means of functional inhibitors of acid sphingomyelinase (FIASMAs), a class of which is represented by the antidepressant fluvoxamine, has emerged as a remarkably effective approach. By blocking the stress-induced hydrolysis of sphingomyelin into pro-apoptotic ceramides, fluvoxamine reduces the formation of ceramide-rich macrodomains on the surfaces of host cells, which serve as viral ports of entry. Observational data shows a statistically significant decrease in the risk of clinical deterioration amongst patients receiving FIASMAs at the onset of infection, suggesting that pharmacological ceramide dampening is a viable, low-cost intervention for the early management of viral pneumonia (227–230).

**Figure 4:**
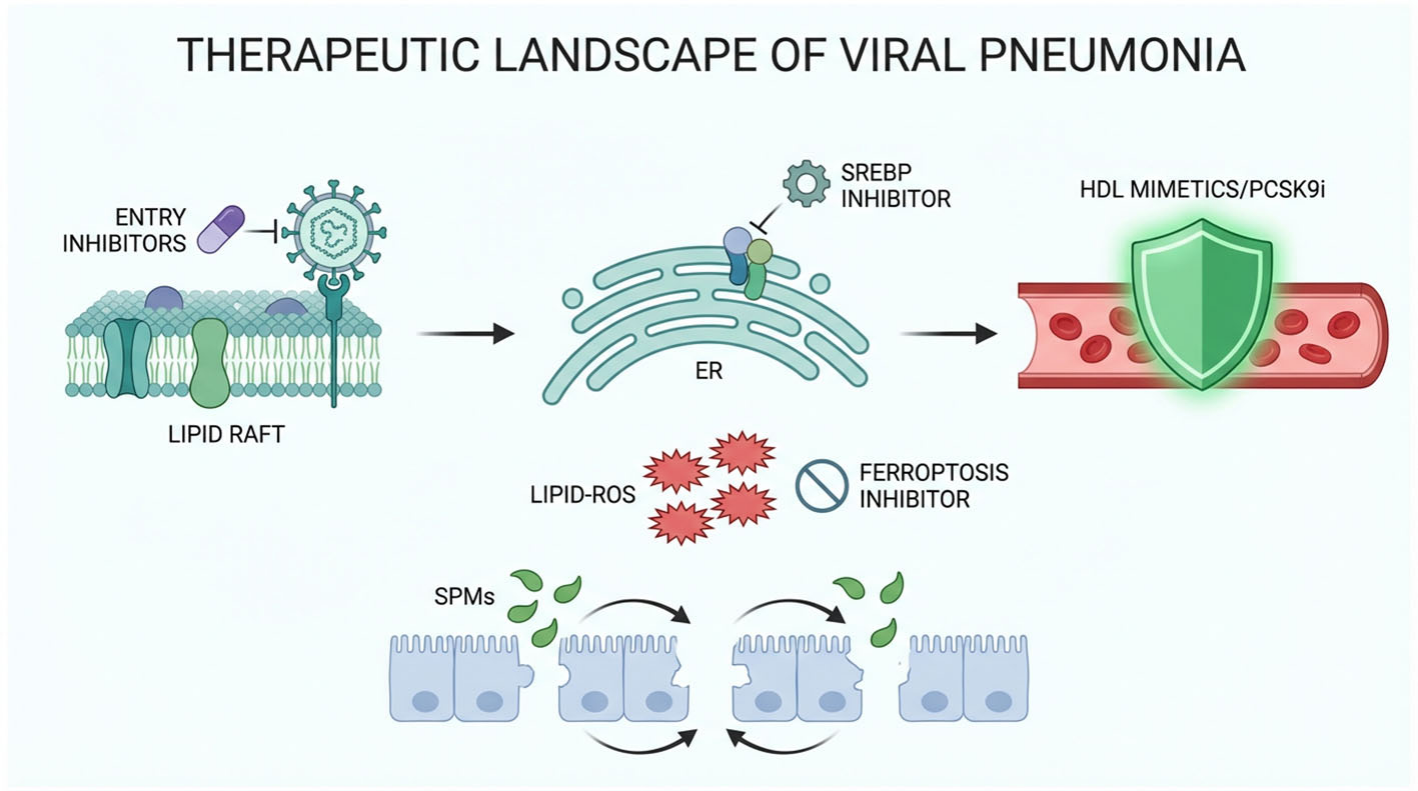
Therapeutic landscape: metabolic-targeted interventions.

A critical caveat is that metabolic interventions operate within narrow temporal windows. SREBP inhibition, while potentially antiviral during the acute phase, may impair surfactant regeneration and delay alveolar repair if sustained into the recovery phase. Likewise, ferroptosis inhibitors, although protective against acute lung injury, could theoretically interfere with physiological cell turnover during tissue repair. Therefore, metabolic therapies require phase-specific application guided by dynamic lipidomic monitoring, rather than fixed, one-size-fits-all regimens (**Figure 4**).

### 5.6 Precision metabolic triage: machine learning and the future of lipid-guided therapy

The ultimate clinical utility of lipidomics lies not in a “one-size-fits-all” supplementation, but in the ability to deliver Precision Metabolic Support based on individual lipidomic profiles. It provides the first blueprint for integrating lipidomic data into the electronic health record (EHR) using Machine Learning (ML) algorithms (231, 232).

Current ML models (predominantly Random Forest and Gradient Boosting Machines) can accurately classify patients into distinct metabolic endotypes. Such endotype-driven stratification holds promise for guiding personalized metabolic support, including targeted antioxidant and lipid-modulating strategies tailored to individual metabolic profiles (233, 234).

The implementation of such a system requires the transition from laboratory-based LC-MS/MS to point-of-care (POC) mass spectrometry—a technology currently under active development. Emerging evidence supports that matching lipidomic profiles to targeted therapeutic agents may enhance the precision of host-directed therapy. This transition from “symptomatic management” to “metabolic correction” represents a key direction in the treatment of severe viral pneumonia and ARDS, offering the potential to advance beyond conventional non-specific immunotherapy in the ICU (22, 75, 76, 235).

## 6 Challenges, controversies, and future perspectives

### 6.1 The causality-correlation dilemma: drivers, passengers, or protective compensators?

A fundamental intellectual challenge in the clinical application of lipidomics relates to the “Causality-Correlation Dilemma.” Current evidence suggests that the “lipid crash,” which is characterized by hypocholesterolemia and depletion of apolipoprotein A-I, is an almost universal hallmark of severe viral pneumonia; however, the scientific community is still divided on whether these perturbations are active contributors to organ failure or merely passive metabolic “passengers” of the severity of the systemic inflammation (23, 236).

To address this uncertainty, the use of Mendelian Randomization (MR) has become the gold standard of causal inference. Recent MR studies by the ISARIC and host genetics consortia have suggested that genetically determined low HDL-cholesterol levels and high triglyceride concentrations are strongly linked with a higher risk for the need for invasive mechanical ventilation (237, 238). These findings provide a strong genetic basis for the hypothesis that differences in the architecture of lipidomic structure predestine host susceptibility. Nevertheless, there is a further controversy about protective compensation: whether elevations of certain lipids, including the transiently increased LPCs, are a pathological expression of membrane breakdown or a host response aimed at recycling the precursors of pulmonary surfactant recovery (22, 239). Future investigations will need to go beyond static observation and use fluxomics, with stable isotope labelling, to measure the actual rates of turnover of lipids throughout the transition from the viremic to the hyper-inflammatory stage. Without a detailed understanding of these fluxes, therapeutic interventions may be associated with suppression of metabolic pathways that are in fact involved in protective host defense (240, 241).

### 6.2 The spatial and single-cell frontier: mapping the alveolar metabolic microenvironment

Traditional “bulk” lipidomics, whether it be from serum or bronchoalveolar lavage fluid (BALF), produces a homogenized average that cannot reflect the extreme spatial heterogeneity of lung parenchyma during viral assault. The next 10 years of research will therefore be defined by the rise of Spatial Lipidomics (MALDI-MSI) and Single-Cell Lipidomics (242–244).

The present data show that the “ferroptotic signature” (e.g. enrichment of PE-AA/AdA) is not homogeneously distributed, but highly concentrated in AECII cells at the site of viral entry, whereas the neighboring capillary endothelial cells might express a completely different signature characterized by depletion of sphingosine-1-phosphate (S1P). Spatial imaging allows these “metabolic hotspots” to be mapped and the exact cellular junctions at which the barrier is broken down to be identified. In addition, the new field of science known as single-cell lipidomics is beginning to uncover “metabolic sub-clusters” of alveolar macrophages. We are now realizing that within the same lung, some of the macrophages are “lipid-congested” (pro-inflammatory M1 phenotype), while others are maintaining a “lipid-efflux” profile (pro-resolving M2 phenotype) (245–247). Deciphering the signals that lead a cell into one of these metabolic states will be important to the development of “cell-specific” lipid stabilizers that can be delivered through inhalation, thereby avoiding the side effects that occur when metabolic drugs are delivered systemically (by mouth, for example) (248, 249).

### 6.3 AI-driven lipidomic signatures: from black-box correlation to mechanistic discovery

The incorporation of machine learning (ML) and artificial intelligence (AI) in lipidomics has come to a critical juncture. While modern ML models, including XGBoost and Random Forest, can predict the probability of mortality from acute respiratory distress syndrome (ARDS) with an AUC >0.90 using only 10 lipid species, these models have been criticized for their opacity and for being uninterpretable from a biological standpoint (250–252).

Literature suggests a move towards explainable AI (XAI), where deep learning architectures are limited by known biochemical pathways. Knowledge-guided neural networks (KGNNs) can not only determine which patients are likely to decline but also explain how, for example, the risk for a given patient is determined by dysfunction in the FADS2-mediated desaturation pathway (not a dietary omega-3 fatty acid deficiency) (253, 254). This transition has potential for the clinical use of point-of-care (POC) lipidomics in the intensive care unit. An integrated AI-POC lipidomics platform that can analyze the rapid mass-spectrometric scans, and provide the personalization of “lipid cocktail” recommendations represents a potential benchmark of precision medicine in the critical care setting, although it has not yet been validated in the clinical setting (76, 255).

### 6.4 Metabolic rehabilitation and the “lipid scarring” of long-COVID

Finally, the long-term consequences of the so-called “lipid crash” require careful consideration. Emerging evidence suggests that the survivors of severe viral pneumonia do not return immediately to their pre-infection metabolic state when they clear the virus, but too often leave with “persistent lipidomic scarring”—a chronic dyslipidemic state which can persist for a year or more and contribute to Long COVID syndrome (256–258).

This persistent state is described by the chronic depletion of the HDL-proteome and a sustained elevation of D-erythro-sphingolipids, biomolecules which have been associated with cognitive fog and chronic fatigue. This observation leads to the provocative hypothesis of metabolic rehabilitation: is it possible that the targeted lipid supplementation or SREBP-resetting therapies, given during the recovery phase, could eliminate these metabolic sequelae (259, 260)? Resolution of pulmonary inflammation is an active and lipid-dependent process requiring a specific metabolic signal. In the absence of such a signal, the lung is in a state of persistent “smoldering” inflammation, culminating eventually in post-viral fibrosis. Investigating the epigenetic-lipid interaction, and in particular how viral infection leaves lasting epigenetic marks on the genes that regulate lipid synthesis, will form the main focus of future Long COVID studies (261–263).

## 7 Concluding remarks: towards a unified immunometabolic theory of ARDS

In conclusion, the path from clinical lipidomic signatures to molecular hijacking and systemic regulation describes a central truth: viral pneumonia represents not only an infectious disorder but a metabolic crisis. The host lipidome is the decisive battleground in which the pathogen’s need for replication organelles is opposed to the host’s need for membrane integrity and control of inflammation.

The shift from the prevailing “one-size-fits-all” approach to a lipid-centric precision medicine framework is underway. By tuning the host metabolic rheostat—re-establishing the immunometabolic shield of high-density lipoprotein or HDL, counteracting the oxidative breach of ferroptosis, and exploiting the resolution potential of specialized pro-resolving mediators or SPMs—we can move beyond blunt force immunosuppression. The future of respiratory medicine hangs on our ability to not only eliminate viral pathogens but to maintain host homeostasis, thereby making the lung’s delicate lipid microenvironment resilient to the challenge of emergent pandemic threats (60, 264, 265).
